# Multiagent off-screen behavior prediction in football

**DOI:** 10.1038/s41598-022-12547-0

**Published:** 2022-05-23

**Authors:** Shayegan Omidshafiei, Daniel Hennes, Marta Garnelo, Zhe Wang, Adria Recasens, Eugene Tarassov, Yi Yang, Romuald Elie, Jerome T. Connor, Paul Muller, Natalie Mackraz, Kris Cao, Pol Moreno, Pablo Sprechmann, Demis Hassabis, Ian Graham, William Spearman, Nicolas Heess, Karl Tuyls

**Affiliations:** 1 DeepMind, London, UK; 2 Liverpool Football Club, Liverpool, UK

**Keywords:** Applied mathematics, Computer science

## Abstract

In multiagent worlds, several decision-making individuals interact while adhering to the dynamics constraints imposed by the environment. These interactions, combined with the potential stochasticity of the agents’ dynamic behaviors, make such systems complex and interesting to study from a decision-making perspective. Significant research has been conducted on learning models for forward-direction estimation of agent behaviors, for example, pedestrian predictions used for collision-avoidance in self-driving cars. In many settings, only sporadic observations of agents may be available in a given trajectory sequence. In football, subsets of players may come in and out of view of broadcast video footage, while unobserved players continue to interact off-screen. In this paper, we study the problem of multiagent time-series imputation in the context of human football play, where available past and future observations of subsets of agents are used to estimate missing observations for other agents. Our approach, called the *Graph Imputer*, uses past and future information in combination with graph networks and variational autoencoders to enable learning of a distribution of imputed trajectories. We demonstrate our approach on multiagent settings involving players that are partially-observable, using the Graph Imputer to predict the behaviors of off-screen players. To quantitatively evaluate the approach, we conduct experiments on football matches with ground truth trajectory data, using a camera module to simulate the off-screen player state estimation setting. We subsequently use our approach for downstream football analytics under partial observability using the well-established framework of pitch control, which traditionally relies on fully observed data. We illustrate that our method outperforms several state-of-the-art approaches, including those hand-crafted for football, across all considered metrics.

## Introduction

Predictive modeling of multiagent behaviors has been a topic of considerable interest in machine learning^1–3^, financial economics^4–6^, robotics^7–9^, and sports analytics^10–13^. In such systems, decision-making agents interact within a shared environment, following an underlying dynamical process that may be stochastic and often infeasible to characterize analytically due to the complex interactions involved. Learning a dynamical model of such systems enables both the understanding and evaluation of agents’ behaviors. Ideally, methods that learn models of such coupled dynamical systems should enable the prediction of future behaviors, the retrodiction of past behaviors, and ultimately the imputation (i.e., filling-in) of partially-occluded data, while respecting any constraints imposed by available observations. In this paper, we introduce such a method for multiagent time-series imputation under temporal occlusion, focusing specifically on the setting of prediction of human football players. In the football domain, off-screen player predictions are crucial for enabling the application of downstream analysis techniques such as pitch control^14^, which rely on information about positions of *all* players in the game.

Football is an especially interesting testbed for the multiagent imputation problem as it involves dynamic interaction of several individuals and stochasticity due to the human decisions involved. A large corpus of prior works have targeted learning models for forward-prediction of multiagent trajectories^10–13,15–19^. In these works, a stream of observations for all involved entities (e.g., all players and the ball) is assumed to be available for some number of timesteps, after which the states of a subset of entities are predicted. However, the availability of full tracking information is a restrictive assumption (requiring the use of proprietary sensors from third-party providers). In many situations, only partial information about the state of a game is available (e.g., positions of only the players visible on broadcast camera), thus requiring imputation of missing data. In contrast to prior works, we target this under-explored multiagent imputation regime, wherein we assume available observations of on-screen players (e.g., as obtained from a vision-based tracking system), and seek to predict the unobserved states of off-screen players, which can subsequently be used for downstream football analytics. Imputation of multivariate time series data involving interacting entities has various practical applications besides football. In financial markets, certain foreign exchange quotations are available more frequently than others, yet correlations between these financial instruments can be used to impute the missing data^4,5,20^. In clinical trials, multi-sensory data may be made with irregular measurements or unavailable for some sensors at certain times^21^. In natural language processing, in-filling of text conditioned on surrounding sentence context is an area of active research^22^, and can naturally extend to multiagent conversational dialogue in-filling.

The partially-observable multiagent trajectories imputation problem is a new regime, which stands in contrast to previous works that only consider forecasting/future-predictions. The key contributions of this paper are as follows. First, we introduce a technique for multiagent imputation, which is applicable even under dynamic occlusion of random subsets of agents in a given trajectory sequence. Our model uses a combination of bidirectional variational LSTMs^23^ and graph networks^24^, with elements in place to handle arbitrarily-complex occlusions of sensory observations in multiagent settings. Second, we illustrate how our approach can be used to extend existing football analytics frameworks (namely, pitch control^14^) to partially-observable settings. Our experiments are conducted on a large suite of 105 full-length real-world football matches, wherein we compare our method against a number of existing approaches including Social LSTMs^7^ and graph variational RNNs (GVRNNs)^3,12^. To our knowledge, this is the first study of trajectory imputation models in the football regime, and bears the potential to unlock the applicability of a vast number of prior analysis techniques (similar to pitch control) to football games that have only intermittent or partially-observable player tracking information.

## Results

This section provides a high-level overview of the proposed approach and empirical results. Readers are referred to the “Methods” section for full technical details of the approach.

### Problem formulation

**Figure 1:**
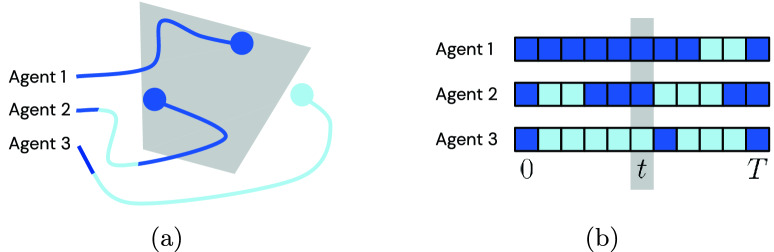
Stylized visualization of the multiagent time-series imputation setting. (**a**) Agent trajectories up to and including time *t*. Dark blue indicates trajectory portions that are observed (with light indicating otherwise); the camera field of view at the current time *t* is indicated in grey. (**b**) Visualization of masks ${{\varvec{m}}}$ for all timesteps, where ${{\varvec{m}}}^i_t=1$ where dark, and ${{\varvec{m}}}^i_t=0$ where light. The mask at time *t*, which corresponds to the frame shown in (**a**), is highlighted in grey.

We first define the multiagent time-series imputation problem, with football as the motivating example. As shown in Fig. 1a, observations of individual players may be unavailable when they are out of the camera frame, and players may disappear and reappear in view multiple times throughout a trajectory sequence. Moreover, the role of any individual player may change multiple times throughout a given trajectory sequence (e.g., a defender can behave in the manner of a midfielder or forward). This characteristic has been well-investigated in prior works^10,12^ and ultimately implies that learned models should be invariant to permutations of player orders within each team. Such models should learn to predict the behavior of players conditioned on the game context, rather than purely on the players’ prescribed roles in the team’s formation.

Regarding notation, we henceforth refer to any scalars associated with an agent *i* at time *t* using unbolded variables, e.g., $s^{i}_{t}$. We use bold notation for vectors (e.g., ${{\varvec{v}}}^{i}_t$). The concatenation of scalars or vectors across time and/or agent indices is denoted by, respectively, dropping the corresponding subscripts and superscripts (e.g., ${{\varvec{s}}}= s^{1:N}_{0:T}$ and ${{\varvec{s}}}_t = {{\varvec{s}}}^{1:N}_t$).

We consider a set of *N* agents ${\mathbb {I}}= \{1,\ldots ,N\}$. Let ${{\varvec{x}}}^{i}_{t} \in \mathbb {R}^{d}$ denote the *d*-dimensional observation of the agent $i \in {\mathbb {I}}$ at time $t \in {\mathbb {T}}= \{0,\ldots , T\}$. In the football scenario considered in our evaluations, $d=2$, with ${{\varvec{x}}}^{i}_t$ corresponding to the (*x*, *y*) position of a player or the ball on the pitch at time *t*. For simplicity, we henceforth refer to ${{\varvec{x}}}^{i}_t$ as the state (rather than observation) of agent *i*, as it comprises the variable of interest we seek to estimate in this work. In the time-series imputation regime, at each time step $t\in {\mathbb {T}}$, observations may be missing for any subset of players. Let ${{\varvec{x}}}= {{\varvec{x}}}^{1:N}_{0:T}$ be observed at the timesteps indicated by an agent-wise masking matrix ${{\varvec{m}}}$ valued in $\{0,1\}^{d}$, such that a given dimension of ${{\varvec{m}}}^{i}_t$ is equal to 1 whenever an observation of agent *i* is available at timestep *t*, and 0 otherwise (see Fig. 1b). In the football context, each player’s on-pitch (*x*, *y*) position is either fully observed at a given time, or fully unobserved (i.e., ${{\varvec{m}}}^{i}_t \in \{(0,0), (1,1)\}$, such that there are no situations where a player’s *x*-position is observed while their *y*-position is not, or vice versa). The objective is then to compute estimates ${\hat{{{\varvec{x}}}}} \in \mathbb {R}^{d}$ of all the unobserved agent states at all timesteps. More precisely, the multiagent time-series imputation problem takes the observed states ${{\varvec{x}}}\odot {{\varvec{m}}}$ as input, where $\odot$ refers to the Hadamard (or element-wise) product, and aims to output a full prediction ${\hat{{{\varvec{x}}}}}^{1:N}_{0:T}$. We quantify this in our experiments via the evaluation loss ${\mathcal {L}}_{2}({\hat{{{\varvec{x}}}}} \odot (1-{{\varvec{m}}}), {{\varvec{x}}}\odot (1-{{\varvec{m}}}))$.

### Workflow

This section provides a high-level overview of the proposed model, called the *Graph Imputer*. For full technical details on the model architecture, hyperparameters, and training, readers are referred to the “Methods” section.

**Figure 2:**
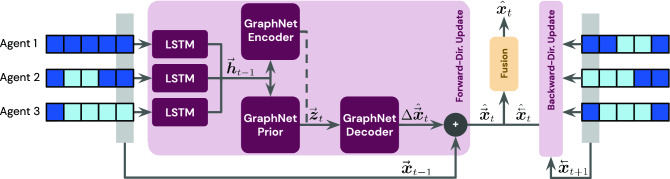
Graph Imputer model. Our model imputes missing information at each timestep using a combination of bidirectional LSTMs and graph networks. An exposition of a forward-direction update (corresponding to directionalupdate in Algorithm 1 in the “Methods” section) is provided in the left portion of the figure. Dark blue boxes indicate trajectory segments that are observed for each agent (with light blue indicating otherwise). In each direction, agent-specific temporal context is updated via LSTMs with shared parameters. All agents’ LSTM hidden states, ${\mathop {{{\varvec{h}}}}\limits ^{{\tiny \rightarrow }}}_{t-1}$, are subsequently used as node features in variational graph networks to ensure information-sharing across agents. This enables learning of a distribution over agent state deviations, $\Delta {\mathop {{{\varvec{x}}}}\limits ^{{\tiny \rightarrow }}}_{t}$. The process is likewise repeated in the backward-direction (right portion of the figure), with the directional updates fused to produce an imputed estimate ${\hat{{{\varvec{x}}}}}_t$ at each time *t*. The dotted line indicates that the Graphnet encoder is used only at training time, with the GraphNet prior being used for the final evaluations conducted at test time.

Figure 2 provides an overview of our proposed approach. The agents modeled in our domain of interest are human football players, who can exhibit stochastic behaviors on-pitch. To enable learning of stochastic predictions given an observation stream, our model learns along two axes: (i) across time via bidirectional LSTMs, which autoregressively generate unobserved agent states; and (ii) across agents via a combination of graph networks (GraphNets)^24^ and variational RNNs (VRNNs)^23^, which model the multiagent interactions involved and enable sampling of distributions of imputed trajectories. The forward- and backward-direction imputed states are fused at each timestep, thus ensuring that all available temporal and agent-interaction information is used throughout the entire generated sequence.

In Fig. 2, we expand the forward-directional update for the agents to provide clearer exposition of the updates conducted; the backward-direction update is analogous, as detailed in “Methods”. Given a stream of observations in each direction, LSTM hidden states are collected across all agents, thus summarizing temporal information; in Fig. 2, we refer to the collection of agents’ hidden states as ${\mathop {{{\varvec{h}}}}\limits ^{{\tiny \rightarrow }}}_{t-1}$ for forward-directional updates. We subsequently use GraphNets to conduct inter-agent information sharing, with each graph being composed of *N* nodes corresponding to the number of agents in the system (e.g., $N = 23$ in football, corresponding to players from both teams and the ball itself). To estimate the distribution of player behaviors, we use a variational approach, relying on a GraphNet encoder, prior, and decoder. The GraphNet encoder and prior take as input the agent hidden states, which are used to initialize their node features; as in typical variational training schemes, the encoder is also provided access to privileged information available only at training-time (in this case, the full ground truth state ${{\varvec{x}}}_t$). The prior, by contrast, does not have access to this information, and is ultimately the model used at evaluation-time. Similar to typical variational approaches, we impose a Kullback-Leibler (KL) divergence term in our training loss to encourage consistency between the encoder and prior distributions (see (15) in our “Methods” section for details).

Following initialization of node features, message passing is conducted within both the GraphNet encoder and prior, which output variables parameterizing the latent distribution. In the message passing step, each graph node (agent) shares information with all other nodes via the graph edges; all node features are then updated given the shared information. Latent distributions are assumed to be Gaussian in our case, as this permits closed-form solution of the KL-divergence term in the training loss. Thus, the parameters output from the GraphNet encoder and prior correspond to the means and covariances of the latent Gaussian distributions. Latent variables are then sampled for each GraphNet node (i.e., agent) from these distributions, which summarize the state of play at the particular timestep *t*; these latent variables are denoted ${\mathop {{{\varvec{z}}}}\limits ^{{\tiny \rightarrow }}}_t$ for the forward-directional update in Fig. 2. The GraphNet decoder subsequently maps these latent variables to estimates of agents’ relative state changes (e.g., $\Delta {\hat{{\mathop {{{\varvec{x}}}}\limits ^{{\tiny \rightarrow }}}}}_{t}$ for forward-direction updates), which are added to the absolute agent states from the previous timestep, ${\mathop {{{\varvec{x}}}}\limits ^{{\tiny \rightarrow }}}_{t-1}$, thus resulting in a directional state estimate ${\hat{{\mathop {{{\varvec{x}}}}\limits ^{{\tiny \rightarrow }}}}}_{t}$. An analogous approach is used for computing a backward-directional state estimate, ${\hat{{\mathop {{{\varvec{x}}}}\limits ^{{\tiny \leftarrow }}}}}_{t}$. These direction-specific predictions are then fused to compute the final bidirectional updates for the agents, ${\hat{{{\varvec{x}}}}}_{t}$. In our ablative experiments (in the Supplementary Information), we test two forms of forward–backward fusion: mean-fusion (simply taking the mean of directional estimates to compute the final prediction) and nearest-weighted fusion (weighing each direction according to the nearest ground truth observation available in that direction). Both of these fusion modes are detailed in the “Methods” section.

Our overall approach autoregressively estimates agent states, using available information in both directions. The model is inherently designed to handle noisy data through two means. First, the bidirectional nature of the model helps ensure it uses information available in future timesteps to correct for such noise. Second, the model is designed to handle noisy data due to its variational nature; namely, the model itself generates noisy autoregressive predictions during its imputation phase, which capture the distribution over input noise, and can thus lead to generation of diverse samples of trajectory outputs. The trajectory sequences generated can subsequently be used for downstream football analytics, as illustrated in our experiments.

### Evaluation

In this section, we empirically evaluate the Graph Imputer against a range of existing models for trajectory prediction.

#### Dataset

We use a dataset of 105 English Premier League matches, where all on-pitch players and the ball are tracked at 25 frames-per-second for each match. We partition the data into trajectory sequences of 240 frames (each capturing 9.6s of gameplay), then downsample the data to 6.25 frames-per-second. For training purposes, we retain only trajectories with 22 players available in the raw data (such that we can compute losses against all players’ ground truth). The data is spatially realigned such that the team in possession always moves towards the right of the pitch (as done in prior works^25^). Finally, for training and evaluation, we split the resulting data into two partitions of 30838 and 4024 trajectories, respectively.

#### Simulated camera model

We use a simulated camera model to generate an observation mask for the task of off-screen player trajectory imputation. The camera model is parameterized by its position and horizontal and vertical field of view angles, with the parameters chosen to produce a vantage point similar to a stadium broadcast camera. For simplicity, the camera-normal is set to track the ball position at each timestep. By intersecting the camera view cone with the pitch plane, we obtain the projected in-frame polygon and mask out-of-frame players accordingly (as in Fig. 1). Further details on the camera model and levels of partial observability imposed due to it are provided in the “Methods” section.

#### Baselines

We compare our approach against the following baselines. *Spline:* Linear, quadratic, and cubic spline interpolation of players’ positions from the moment they leave the the camera field of view to the moment they return; these approaches are simple, though can exhibit reasonable performance as they ensure the predicted trajectories adhere to the boundary value constraints imposed by the last observation of each player prior to going off-screen, and their first observation upon re-emergence on-screen. *Autoregressive LSTMs:* A simple baseline using autoregressive LSTMs, run independently per player for state estimation. *Role-invariant VRNNs:* A strong variational baseline that we hand-craft for the football scenario (i.e., assuming two teams of an equal number of players), using VRNNs and a combination of post-processing steps to ensure information-sharing between players on each team, and invariance of model outputs to re-ordering of players in inputs. Refer to the Additional Experiment Details section of the Supplementary Information for further information. *Social LSTM*^7:^ A model that uses ‘social pooling’, which is a technique that pools hidden states of neighboring agents to ensure spatially-nearby context is appropriately shared between individual agents. *Bidirectional Social LSTMs:* We also implement a bidirectional Social LSTM variant using a combination of the vanilla Social LSTM updates and the fusion Eqs. (1), (2), (13) and (14) detailed in our “Methods” section, which we have not observed being used in the literature for our problem regime. *GVRNNs*^12:^ A model that uses a combination of unidirectional VRNNs and Graph Neural Networks (similar in nature to Graph-VRNNs^3^), but assumes full observability of players for a fixed number of timesteps and targets the forward-prediction setting.

**Table 1:** Football off-screen player state estimation results.

	Model	Skip connection	Next-step conditional decoder	${\mathcal {L}}_{2}(\text {Mean})$		${\mathcal {L}}_{2}(\text {Min.})$	
Restricted	Role-invariant VRNN	✗	−	2.020	2.03	1.960	2.063
	Role-invariant VRNN	✓	−	0.958	0.009	0.953	0.009
	Bidir. Role-invariant VRNN	✗	−	0.174	0.002	0.160	0.002
	Bidir. Role-invariant VRNN	✓	−	0.167	0.002	0.166	0.002
General	Spline (linear)	−	−	0.658	0.081	−	
	Spline (Quadratic)	−	−	0.197	0.023	−	
	Spline (Cubic)	−	−	0.193	0.023	−	
	LSTM	−	−	1.579	0.019	−	
	Bidir. LSTM	−	−	0.350	0.006	−	
	Social LSTM^7^	−	−	1.049	0.274	−	
	Bidir. Social LSTM	−	−	0.198	0.052	−	
	GVRNN^12^	✗	✗	2.243	0.136	1.453	0.073
	GVRNN^12^	✗	✓	2.447	1.197	2.400	1.231
	GVRNN^12^	✓	✗	0.882	0.009	0.874	0.009
	GVRNN^12^	✓	✓	0.865	0.018	0.852	0.017
	Graph Imputer (Ours)	✗	✗	0.241	0.05	0.224	0.051
	Graph Imputer (Ours)	✗	✓	0.404	0.102	0.397	0.11
	Graph Imputer (Ours)	✓	✗	0.165	0.005	0.163	0.005
	Graph Imputer (Ours)	✓	✓	**0.153**	**0.003**	**0.151**	**0.003**

We separate models into two categories: restricted models (those that apply only to the football setting, as they process data in a manner explicitly assuming two teams of players, along with a ball), and general models (models that apply to general multiagent prediction settings). The remaining columns refer to the following: *Skip connection* indicates whether a skip-connection from the input to the decoder is enabled for autoencoder based models. *Next-step conditional decoder* indicates whether decoders in graph network-based models condition on available next-timestep observations, as additional context. For each baseline model, we compute the mean evaluation loss, ${\mathcal {L}}_{2}(\text {Mean})$, compared to the ground truth trajectories (over all seeds). For stochastic models, for each evaluation sequence we also take 6 samples of imputed trajectories, and also report the minimum evaluation loss, ${\mathcal {L}}_{2}(\text {Min.})$, over all samples, averaged over all seeds. Best results are in bold.

**Figure 3:**
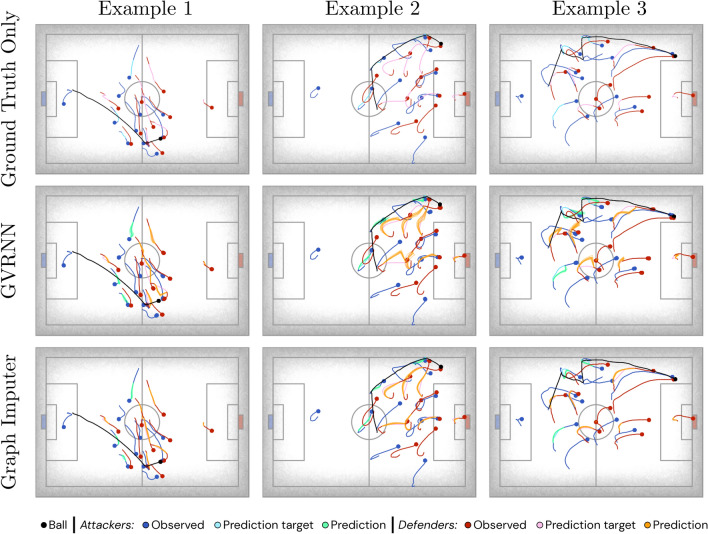
Trajectory visualizations (best viewed when zoomed in). Each column provides an example trajectory sequence, with the first row illustrating the ground truth, and subsequent rows showing results from various models, including the Graph Imputer (ours). For all examples, the Graph Imputer trajectories seamlessly adhere to the boundary value constraints imposed at the moments of disappearance and reappearance of players.

#### Trajectory prediction analysis

Table 1 provides a summary of results for the football off-screen player data imputation regime, including ablations over key model features where applicable. Training and hyperparameter details are provided in the “Methods” section. As noted earlier, the role-invariant models (listed in the first several table rows) are hand-crafted for the football case, and thus are not applicable to general multiagent settings; nonetheless, these models pose a strong evaluation baseline, and outperform several of the more generic approaches. Our proposed model, the Graph Imputer, outperforms the baselines both in terms of the mean and minimum evaluation loss over prediction samples, including the hand-crafted models.

As evident from Table 1, bidirectionality naturally yields a significant improvement in terms of overall performance across the models, as both past and future information is used in estimating player positions when off-screen. This is quantitatively evident even for the linear spline baseline, which is effectively bidirectional as it interpolates the last appearance and first reappearance of each player. While quadratic and cubic interpolation increase performance compared to the linear baseline, our model outperforms them significantly. Despite the additional context provided by past and future observations, such interpolation methods have no understanding of the dynamics of the domain (i.e., football in this case). As such, they can particularly suffer from a decrease in accuracy in situations where off-screen players behave defensively or offensively, exhibit sudden movements, or are off-screen for extended periods of time, which is not well-captured by interpolation. We also anticipate that situations with increased partial observability will further compound these issues with standard interpolation techniques. To further investigate the effects of increased partial observability, we generated a new dataset to test the sensitivity of the models to such changes (see the Sensitivity to Observability Model section in the Supplementary Information for numerical results, which illustrate the robustness of our model to these factors).

Considering the more complex baselines in Table 1, we note that the unidirectional Social LSTM model is outperformed by the strongest-performing GVRNN model (which uses both a skip connection and next-step conditioned graph decoder), as observed in earlier literature. However, as the Social LSTM is fundamentally deterministic in nature, it cannot be used for sampling multiple viable player trajectories. We additionally observe that use of a skip connection from inputs to the decoder results in a significant improvement in results, for all variational models considered. While use of a next-step conditioned graph decoder slightly improves results for the Graph Imputer, it has a more significant impact on the GVRNN model, which we conjecture is due to the former model’s bidirectional nature already providing significant information about future observations.

Figure 3 provides static visualizations of trajectory results for several example sequences, with more examples included in the Additional Experiment Results section of the Supplementary Information. We recommend readers view our animated visualizations on our website (https://sites.google.com/view/imputation-of-football/), which also illustrate the simulated camera model. In Fig. 3, observed trajectory segments for the attacking and defending team are, respectively, illustrated in dark blue and red, with the ball trajectory indicated in black. In the first row of the figure, we illustrate the portion of player trajectories that are unobserved in light blue and pink for each team, respectively. Recall that the observations provided to the models are the raw positions available for in-camera players, with the camera tracking the ball in each timestep. Well-performing models will, ideally, learn the key behavioral characteristics of player interactions and physics (e.g., velocities, constraints on acceleration, player turning radii, etc.) given the available positional information to make realistic predictions. The subsequent rows illustrate the predictions made by both the GVRNN model and the Graph Imputer, under the same observations. Notably, the bidirectional nature of our Graph Imputer approach enables predictions to not only more accurately model the flow of movement of players on the pitch, but also to appropriately adhere to the boundary value constraints imposed by players when they appear back in the camera view. For additional experiments, including numerous visualizations over baseline models and ablations over the bidirectional fusion modes discussed in “Methods”, refer to the Additional Experiment Results section of the Supplementary Information.

#### Pitch control analysis

A key benefit of our model is that it can enable the downstream use of well-established football analytics models that rely on full player tracking information. In situations where this is infeasible (e.g., where player tracking information is obtained from broadcast video footage, as opposed to proprietary sensors installed in stadiums), such downstream models can no longer be applied. However, through the imputation of off-screen player trajectories, our approach enables the applicability of such models. Moreover, due to the bidirectional nature of our model, the predicted trajectories are significantly more useful for post-hoc analytics in comparison to standard forward-predictive approaches. To illustrate this, here we run experiments evaluating the performance of our approach when used to compute pitch control^14^, which is a well-known model used for trajectory-based football analytics. Pitch Control, at a high-level, is a technique for computing the probability of each player (or team) gaining control of the ball were it to be passed to any location on pitch. For details of the pitch control model itself, we refer readers to the “Methods” section.

**Table 2:** Predicted vs. ground truth pitch control^14^ Mean Average Error (MAE) across models, under partially observable settings.

	Model	Pitch control MAE
Restricted	Role-invariant VRNN	0.0447 ± 0.0204
	Bidir. Role-invariant VRNN	0.0209 ± 0.0096
General	LSTM	0.0592 ± 0.0274
	Bidir. LSTM	0.0296 ± 0.0139
	Social LSTM^7^	0.0474 ± 0.0216
	Bidir. Social LSTM	0.0219 ± 0.0098
	GVRNN^12^	0.0418 ± 0.0189
	Graph Imputer (Ours)	0.0207 ±0.0094

Mean and standard deviations are reported over all trajectories in our validation dataset. The Graph Imputer model yields the lowest pitch control error across all baselines. Note that the Bidir. Role-invariant VRNN model, which comes closest to our Graph Imputer model in terms of performance, was handcrafted by us specifically for the football domain.

**Figure 4:**
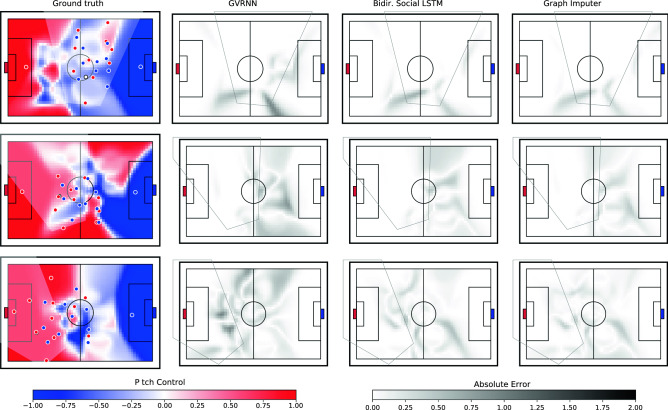
Pitch control error visualizations. The first column shows the ground truth pitch control field, player positions, and the camera field of view. Each remaining column provides a visualization of the absolute error between pitch control fields based on predicted model outputs and ground truth.

To compare models in terms of application to downstream analytics, we first compute the ground truth pitch control *without* any partial observability for all trajectories in our validation dataset. This results in a pitch control field at each timestep of each trajectory, which is a scalar field over the pitch (see examples in Fig. 4—left column). We subsequently consider pitch control under partial observability by occluding player positions as in our previous experiments, generate imputed trajectories using the various trained models, and compute model-specific pitch control fields. We then compute the Mean Absolute Error (MAE) between the ground truth and predicted Pitch Control fields, averaging spatially (across the pitch) and temporally (along the entire trajectory sequence). We report the pitch control MAE across all trajectories in Table 2. Notably, our Graph Imputer model yields the lowest error across all baselines. While the Bidirectional Role-invariant VRNN model comes close in terms of performance, we note that this was also a model carefully handcrafted by us for the football setting. Given its generality, the performance of the Graph Imputer is notable here.

To better understand differences qualitatively, in Fig. 4 we visualize several examples of the predicted and ground truth pitch control fields. Each row of the figure corresponds to a distinct game scenario from our validation dataset. The left column visualizes ground truth player positions for both teams (blue and red points), the camera field-of-view (semi-transparent white overlay), and the ground truth pitch control field. The remaining columns visualize the absolute error between pitch control fields based on predicted model outputs and ground truth. The example in the first row involves a scenario where the majority of players are visible within the camera field-of-view. As such, the pitch control predictions from the GVRNN, Bidirectional Social LSTM, and Graph Imputer are fairly consistent. Nonetheless, the region of the pitch corresponding to the blue team’s goalkeeper (towards the right side of the pitch) has notably higher Pitch control error for the GVRNN compared to the latter models. Such a mis-estimation can have quite detrimental side-effects if used for tactical decision-making in games, especially as determination of pitch control in regions near each goalkeepers is crucial for determining goal-scoring opportunities (as illustrated in Spearman^26^). The example in the second row involves a larger number of off-screen players. Here we can see a noticeably high error in the GVRNN model near the bottom-right region of the pitch. Moreover, the Bidirectional Social LSTM model also has higher error than ours near the top region of the pitch. The third example further exacerbates these errors, involving a situation where the camera is pointed towards the far left side of the pitch, with half of the players not visible on-screen. Here we see not only a high pitch control error for the GVRNN, but even regions with particularly high error *within the camera field-of-view* for the Bidirectional Social LSTM (see regions directly in front of the red goalkeeper). Such levels of error in on-screen regions are not prominent in the Graph Imputer model, which instead exhibits slightly larger error than the Bidirectional Social LSTM in a small region towards the bottom-right of the pitch. For interested readers, we additionally include animated visualizations of Pitch Control fields on our submission website.

Overall, our evaluations are indicative of the Graph Imputer model’s performance not only in terms of raw trajectory prediction, but also for downstream use-cases such as pitch control-based analysis.

## Related work

There exist a number of works from various fields of research that are related to our approach. Specifically, a number of works from robotics and computer vision^7–9,27^, sports analytics^11–13,16,18^, economics^4–6^ and machine learning^1–3^ focus on various combinations of missing data imputation and multiagent trajectory predictions. Given the broad scope of time series prediction as a research field^28^, we focus particularly on models that predict human trajectories^9^, as they are the most relevant for our problem regime. We also provide a table summarizing key similarities and differences of the most-closely related models in the Additional Details on Related Works section of the Supplementary Information.

One of the most common applications within human trajectory prediction (albeit not directly related to sports), is pedestrian modeling^7,29^. More closely related to our work are models that predict the trajectories of athletes in a team, such as basketball^16,17,19,30^ or football^10–12^. Efforts that focus on the latter vary in the way they treat the interactions between players. While some models directly use the information about the players as conditioning for imitation learning^10,11^, others use more complex interaction models such as graph networks for forward-prediction^3,12^. Our approach builds on the related works of Yeh et al.^12^ and Sun et al.^3^, which operate in the regime of predicting forward-rollouts of trajectories. As noted in Yang et al.^31^ and Shang et al.^32^, the models in Sun et al.^3^ and Yeh et al.^12^ are quite similar to one another, combining Graph Networks with VRNNs. In our comparisons and baselines table, we cite Yeh et al.^12^ as we followed their implementation details most closely. Nonetheless, the problem setting considered by Sun et al.^3^ and Yeh et al.^12^ is quite different from ours. They consider settings involving forward-rollouts of agent trajectories (i.e., observations of agents are made for some number of timesteps, and forward forecasts are conducted thereafter). Our setting, by contrast, involves interacting agents that pop in and out of view sporadically, and bidirectional use of temporal information at test-time, which has not been considered in past works to our knowledge. The main limitation of prior works in application to this regime is that their forward-rollout predictions deviate over time, and thus do not adhere to the constraints imposed by future ground truth observations. An analogous comparison would be Kalman Filtering (using only past observations) versus Kalman Smoothing (using both past and future observations); these share core similarities, but are both independently useful in entirely different settings.

Mohamed et al.^33^, Salzmann et al.^34^ focus on forward-predictions of pedestrian trajectories. Mangalam et al.^35^ also considers future-trajectory prediction, by first learning a distribution of endpoints and subsequently sampling from it. Kipf et al.^36^ focus on forward-predictions of interacting physical systems, while Graber and Schwing^37^ uses backward-information to update the approximate posterior during training, it still targets the forward-prediction regime at test-time (e.g., as noted in Fig. 8 of Graber and Schwing^37^). Thus, as mentioned earlier, these prior works consider only future predictions and not the imputation regime considered in our paper, which we believe is worthy of independent investigation. Finally, despite being framed as a supervised learning problem rather than sequence prediction, Hoshen^15^ also take into account the interactions between the different variables in their multivariate trajectory prediction problem by using interaction networks.

Rather than targeting the forward-prediction regime, the goal of our model is to carry out imputation of incomplete time series involving multiple interacting agents. Imputation of sequential data itself can be treated as a means to an end for a separate task such as classification^38^. Also related to our line of work is prior work on a bidirectional model that carries out trajectory imputation^39^. Unlike ours, however, their approach does not target specifically the multiagent setting, though applies to the regime by essentially treating it as a large single-agent scenario. Finally, approaches that focus more directly on the imputation task itself include GAN-based models^40,41^ and bidirectional inference models^42,43^.

## Conclusion

In this paper, we considered the problem regime of multiagent time-series imputation, which has not been formally analyzed in the literature, in contrast to the forward-predictive regime that has been significantly studied. Our introduced approach, called the Graph Imputer, uses a combination of bidirectional recurrent models to ensure use of all available temporal information, and graph networks to model inter-agent relations. Our experiments focused on the football analytics regime to illustrate a practical and intuitive real-world use case of such models. We illustrated that our approach outperforms several state-of-the-art methods on a large dataset of football tracking data, and qualitatively yields trajectory samples that capture player interactions and adhere to the constraints imposed by available observations. We subsequently illustrated how our approach can be used for unlocking downstream application of more complex analytical tools, such as Pitch control^14^, which have traditionally relied on availability of fully-observed data. Empirical results illustrated that our approach outperforms strong baselines both in terms of raw trajectory prediction performance, and also in terms of these latter football-specific metrics, even outperforming models specifically handcrafted for the football regime.

One of the limitations of our model is that the forward and backward-direction latent vectors, ${\mathop {{{\varvec{z}}}}\limits ^{{\tiny \rightarrow }}}$ and ${\mathop {{{\varvec{z}}}}\limits ^{{\tiny \leftarrow }}}$, are sampled independently in our model; sampling these from a joint underlying distribution could significantly improve correlations in the directional predictions. Moreover, our model requires observations of each agent for at least a single timestep throughout each trajectory. While this is not a major limitation given long enough trajectory sequences in practice, investigating a means of enabling the model to seamlessly handle *completely missing* agents would increase its generality. An additional improvement to the model could be incorporation of distributional information (namely, the predicted covariance matrices) in the bidirectional fusion methods used, leveraging ideas from existing information filtering techniques. Finally, note that the training dataset constructed in our approach replicates the setup of related approaches (e.g.^12^). The diversity of behaviors expressed even within a single game makes the prediction problem challenging even with such a dataset split. Nonetheless, it may be interesting in future work to consider transfer learning situations (involving training on one set of games, and evaluating on a hold-out set), which are likely to be of interest in situations involving vision-in-the-loop (e.g., where camera systems are used to track players with varying uniforms, across different leagues).

Overall, the key benefit of our approach is its generality, in the sense that it permits any subset of agents to be unobserved at any timestep, works with temporal occlusions of arbitrary time horizons, and can apply directly to general multiagent domains beyond football. Predictive modeling of human trajectories is a complex problem with numerous applications, such as sports analytics, pedestrian modeling on roads, and crowd modeling in stadia. Given the fairly general nature of our approach, a key avenue of future work will be to apply it to these related regimes of predictive modeling.

## Methods

This section provides technical details of our approach, called the Graph Imputer. All methods and research were performed in accordance with relevant guidelines/regulations of Nature Research journals. Algorithm 1 provides the associated pseudocode, and Fig. 2 illustrates the approach at a high level. We next define the specific components of our model in detail.

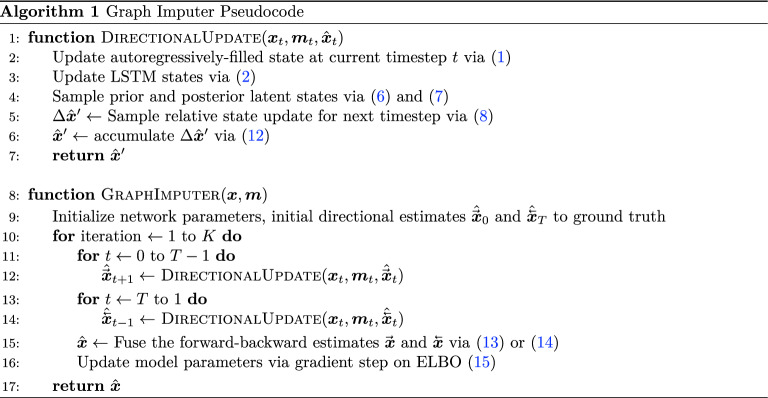



### Bidirectional autoregression

The key distinction between our problem regime and that of many prior multiagent predictive modeling approaches is that we target the more general imputation setting, involving both future and past contextual information about subsets of various agents. The temporal backbone of our model is, thus, a bidirectional autoregressive LSTM, which leverages all available information at the time of prediction.

Specifically, at each time *t*, let ${\mathop {{{\varvec{x}}}}\limits ^{{\tiny \rightarrow }}}_t$ and ${\mathop {{{\varvec{x}}}}\limits ^{{\tiny \leftarrow }}}_t$ denote the forward- and backward-direction inputs to the model. These inputs correspond to the combination of ground truth states, ${{\varvec{x}}}_t$, for observed agents, and autoregressively-predicted states, ${\hat{{\mathop {{{\varvec{x}}}}\limits ^{{\tiny \rightarrow }}}}}_t$ and ${\hat{{\mathop {{{\varvec{x}}}}\limits ^{{\tiny \leftarrow }}}}}_{t}$ (defined below), for unobserved agents, as follows:

1 $$\begin{aligned} {\mathop {{{\varvec{x}}}}\limits ^{{\tiny \rightarrow }}}_t&= {{\varvec{x}}}_t \odot {{\varvec{m}}}_t + {\hat{{\mathop {{{\varvec{x}}}}\limits ^{{\tiny \rightarrow }}}}}_t \odot (1-{{\varvec{m}}}_t) \quad&{\mathop {{{\varvec{x}}}}\limits ^{{\tiny \leftarrow }}}_t&= {{\varvec{x}}}_t \odot {{\varvec{m}}}_t + {\hat{{\mathop {{{\varvec{x}}}}\limits ^{{\tiny \leftarrow }}}}}_t \odot (1-{{\varvec{m}}}_t). \end{aligned}$$

We use bidirectional LSTMs to temporally-integrate observation sequences and learn the forward- and backward-dynamics involved. Agent-wise hidden states, ${\mathop {{{\varvec{h}}}}\limits ^{{\tiny \rightarrow }}}_{t}^{i}$ and ${\mathop {{{\varvec{h}}}}\limits ^{{\tiny \leftarrow }}}_{t}^{i}$, are updated as follows:

2 $$\begin{aligned} {\mathop {{{\varvec{h}}}}\limits ^{{\tiny \rightarrow }}}_t^i = \text {LSTM}_{{\mathop {{{\varvec{\omega }}}}\limits ^{{\tiny \rightarrow }}}}({\mathop {{{\varvec{x}}}}\limits ^{{\tiny \rightarrow }}}_{t}^{i}, {\mathop {{{\varvec{h}}}}\limits ^{{\tiny \rightarrow }}}^{i}_{t-1}) \qquad \qquad \qquad {\mathop {{{\varvec{h}}}}\limits ^{{\tiny \leftarrow }}}_{t}^{i} = \text {LSTM}_{{\mathop {{{\varvec{\omega }}}}\limits ^{{\tiny \leftarrow }}}}({\mathop {{{\varvec{x}}}}\limits ^{{\tiny \leftarrow }}}_{t}^{i}, {\mathop {{{\varvec{h}}}}\limits ^{{\tiny \leftarrow }}}^{i}_{t+1})\, \quad \forall i \in {\mathbb {I}}, \end{aligned}$$

where ${\mathop {{{\varvec{\omega }}}}\limits ^{{\tiny \rightarrow }}}$ and ${\mathop {{{\varvec{\omega }}}}\limits ^{{\tiny \leftarrow }}}$ refer to direction-specific LSTM parameters, which are shared across agents.

We next detail the computation of the autoregressively-predicted states ${\hat{{\mathop {{{\varvec{x}}}}\limits ^{{\tiny \rightarrow }}}}}$ and ${\hat{{\mathop {{{\varvec{x}}}}\limits ^{{\tiny \leftarrow }}}}}$ appearing in (1), which are sampled from a variational graph network capturing multiagent interactions in the system.

### Graph networks

We define a graph network consisting of *N* nodes, each corresponding to an agent or entity in the system (e.g., $N = 23$ in the football domain, capturing the state of the 22 players and the ball). Let ${{\varvec{v}}}^i_t$ denote the node feature vector associated with an agent $i \in {\mathbb {I}}$, which encodes its spatiotemporal context at time *t*. Likewise, let ${{\varvec{e}}}^{(i,j)}$ denote the directed edge feature connecting agent $i \in {\mathbb {I}}$ to agent $j \in {\mathbb {I}}$. Graph networks operate via rounds of message passing, which update edge and node features to propagate information across the various nodes involved. In our instance, the message-passing update is expressed as follows,

3 $$\begin{aligned} {{\varvec{e}}}'^{(i,j)}&= f_{{{\varvec{\theta }}}}^e({{\varvec{v}}}^i, {{\varvec{v}}}^j) \quad&\text {(Update edges from sender nodes }i \in N^{-}(j)\text { to recipients }j \in {\mathbb {I}}), \end{aligned}$$

4 $$\begin{aligned} {{\varvec{e}}}'^j&= \sum _{i \in N^{-}(j)} {{\varvec{e}}}'^{(i,j)} \quad&\text {(Aggregate incoming edges for all receiver nodes }j \in {\mathbb {I}}), \end{aligned}$$

5 $$\begin{aligned} {{\varvec{v}}}'^{j}&= f_{{{\varvec{\theta }}}}^v({{\varvec{e}}}'^j) \quad&\text {(Update all receiver nodes }j \in {\mathbb {I}}), \end{aligned}$$

where $N^{-}(j)$ are in-neighbors of node *j*, and $f_{{{\varvec{\theta }}}}^e$ and $f_{{{\varvec{\theta }}}}^v$ are, respectively, edge and node update functions with learned parameters ${{\varvec{\theta }}}$. In shorthand, given an initial set of node features ${{\varvec{v}}}$, we refer to the updated features following the message-passing steps in (3) to (5) as ${{\varvec{v}}}' = \text {GN}_{{{\varvec{\theta }}}}({{\varvec{v}}})$.

### Variational updates

At any time *t*, the history of autoregressively-filled directional inputs, ${\mathop {{{\varvec{x}}}}\limits ^{{\tiny \rightarrow }}}_{<t}$ and ${\mathop {{{\varvec{x}}}}\limits ^{{\tiny \leftarrow }}}_{>t}$, is encoded by the LSTM states ${\mathop {{{\varvec{h}}}}\limits ^{{\tiny \rightarrow }}}_{t-1}$ and ${\mathop {{{\varvec{h}}}}\limits ^{{\tiny \leftarrow }}}_{t+1}$. Conditioned on this context, our model uses variational graph networks to enable information-sharing across agents, and learn a distribution over latent random variables ${{\varvec{z}}}$ and predicted state updates $\Delta {\hat{{{\varvec{x}}}}}_t$. Specifically, the graph imputer learns to approximate the directional prior distributions $p_{{{\varvec{\theta }}}}({\mathop {{{\varvec{z}}}}\limits ^{{\tiny \rightarrow }}}^i_t|\cdot )$ and $p_{{{\varvec{\theta }}}}({\mathop {{{\varvec{z}}}}\limits ^{{\tiny \leftarrow }}}^i_t|\cdot )$, posterior distributions $q_{{{\varvec{\phi }}}}({\mathop {{{\varvec{z}}}}\limits ^{{\tiny \rightarrow }}}^i_t|\cdot )$ and $q_{{{\varvec{\phi }}}}({\mathop {{{\varvec{z}}}}\limits ^{{\tiny \leftarrow }}}^i_t|\cdot )$, and decoded output distribution $p_{{{\varvec{\theta }}}}(\Delta {\hat{{\mathop {{{\varvec{x}}}}\limits ^{{\tiny \rightarrow }}}}}^i_t|\cdot )$ and $p_{{{\varvec{\theta }}}}(\Delta {\hat{{\mathop {{{\varvec{x}}}}\limits ^{{\tiny \leftarrow }}}}}^i_t|\cdot )$, as follows,

6 $$\begin{aligned} p_{{{\varvec{\theta }}}}({\mathop {{{\varvec{z}}}}\limits ^{{\tiny \rightarrow }}}_t^i | {\mathop {{{\varvec{x}}}}\limits ^{{\tiny \rightarrow }}}_{<t}, {\mathop {{{\varvec{z}}}}\limits ^{{\tiny \rightarrow }}}_{<t})&= {\mathcal {N}}\left( {\mathop {{{\varvec{\mu }}}}\limits ^{{\tiny \rightarrow }}}_{\text {pri},t}^i, ({\mathop {{{\varvec{\sigma }}}}\limits ^{{\tiny \rightarrow }}}_{\text {pri},t}^{i})^2 \right) &p_{{{\varvec{\theta }}}}({\mathop {{{\varvec{z}}}}\limits ^{{\tiny \leftarrow }}}_t^i | {\mathop {{{\varvec{x}}}}\limits ^{{\tiny \leftarrow }}}_{>t}, {\mathop {{{\varvec{z}}}}\limits ^{{\tiny \leftarrow }}}_{>t})&= {\mathcal {N}}\left( {\mathop {{{\varvec{\mu }}}}\limits ^{{\tiny \leftarrow }}}_{\text {pri},t}^i, ({\mathop {{{\varvec{\sigma }}}}\limits ^{{\tiny \leftarrow }}}_{\text {pri},t}^{i})^2 \right) , \end{aligned}$$

7 $$\begin{aligned} q_{{{\varvec{\phi }}}}({\mathop {{{\varvec{z}}}}\limits ^{{\tiny \rightarrow }}}_t^i | {{\varvec{x}}}_t, {\mathop {{{\varvec{x}}}}\limits ^{{\tiny \rightarrow }}}_{< t}, {\mathop {{{\varvec{z}}}}\limits ^{{\tiny \rightarrow }}}_{<t})&= {\mathcal {N}}\left( {\mathop {{{\varvec{\mu }}}}\limits ^{{\tiny \rightarrow }}}_{\text {enc},t}^i, ({\mathop {{{\varvec{\sigma }}}}\limits ^{{\tiny \rightarrow }}}_{\text {enc},t}^{i})^2 \right)&q_{{{\varvec{\phi }}}}({\mathop {{{\varvec{z}}}}\limits ^{{\tiny \leftarrow }}}_t^i | {{\varvec{x}}}_t, {\mathop {{{\varvec{x}}}}\limits ^{{\tiny \leftarrow }}}_{> t}, {\mathop {{{\varvec{z}}}}\limits ^{{\tiny \leftarrow }}}_{>t})&= {\mathcal {N}}\left( {\mathop {{{\varvec{\mu }}}}\limits ^{{\tiny \leftarrow }}}_{\text {enc},t}^i, ({\mathop {{{\varvec{\sigma }}}}\limits ^{{\tiny \leftarrow }}}_{\text {enc},t}^{i})^2 \right) , \end{aligned}$$

8 $$\begin{aligned} p_{{{\varvec{\theta }}}}(\Delta {\hat{{\mathop {{{\varvec{x}}}}\limits ^{{\tiny \rightarrow }}}}}_t^i | {\mathop {{{\varvec{x}}}}\limits ^{{\tiny \rightarrow }}}_{<t}, {\mathop {{{\varvec{z}}}}\limits ^{{\tiny \rightarrow }}}_{\le t})&= {\mathcal {N}}\left( {\mathop {{{\varvec{\mu }}}}\limits ^{{\tiny \rightarrow }}}_{\text {dec},t}^i, ({\mathop {{{\varvec{\sigma }}}}\limits ^{{\tiny \rightarrow }}}_{\text {dec},t}^{i})^2 \right)&p_{{{\varvec{\theta }}}}(\Delta {\hat{{\mathop {{{\varvec{x}}}}\limits ^{{\tiny \leftarrow }}}}}_t^i | {\mathop {{{\varvec{x}}}}\limits ^{{\tiny \leftarrow }}}_{>t}, {\mathop {{{\varvec{z}}}}\limits ^{{\tiny \leftarrow }}}_{\ge t})&= {\mathcal {N}}\left( {\mathop {{{\varvec{\mu }}}}\limits ^{{\tiny \leftarrow }}}_{\text {dec},t}^i, ({\mathop {{{\varvec{\sigma }}}}\limits ^{{\tiny \leftarrow }}}_{\text {dec},t}^{i})^2 \right) . \end{aligned}$$

In the above, (6) enables sampling of latent variables, ${\mathop {{{\varvec{z}}}}\limits ^{{\tiny \rightarrow }}}_t$ and ${\mathop {{{\varvec{z}}}}\limits ^{{\tiny \leftarrow }}}_t$, conditioned on the prior information available up to, though not including, the prediction timestep *t*. Likewise, (7) captures the posterior latent state distribution, conditioned on the same information as the prior *in addition to* the ground truth state ${{\varvec{x}}}_t$. Finally, (8) enables sampling of a next-state prediction for each direction. As in typical VRNN-based approaches, the encoder is used only during training to sample latent states ${{\varvec{z}}}_t$, which are used as inputs for the decoder; during evaluation, samples ${{\varvec{z}}}_t$ from the prior are used instead, as the encoder can, naturally, no longer be used due to the ground truth state ${{\varvec{x}}}_t$ being unavailable.

The collection of mean and variance parameters above, ${{\varvec{\mu }}}$ and ${{\varvec{\sigma }}}^2$, parameterize underlying Gaussian distributions. These parameters simply correspond to node features output by underlying graph networks, which exchange information between agents following a message-passing step:

9 $$\begin{aligned} \left[ {\mathop {{{\varvec{\mu }}}}\limits ^{{\tiny \rightarrow }}}_{\text {pri},t}^i, ({\mathop {{{\varvec{\sigma }}}}\limits ^{{\tiny \rightarrow }}}_{\text {pri},t}^{i})^2\right] _{i \in {\mathbb {I}}}&= \text {GN}_{\text {pri},{{\varvec{\theta }}}}\!\left( {\mathop {{{\varvec{h}}}}\limits ^{{\tiny \rightarrow }}}_{t-1}\right) &\left[ {\mathop {{{\varvec{\mu }}}}\limits ^{{\tiny \leftarrow }}}_{\text {pri},t}^i, ({\mathop {{{\varvec{\sigma }}}}\limits ^{{\tiny \leftarrow }}}_{\text {pri},t}^{i})^2\right] _{i \in {\mathbb {I}}}&= \text {GN}_{\text {pri},{{\varvec{\theta }}}}\!\left( {\mathop {{{\varvec{h}}}}\limits ^{{\tiny \leftarrow }}}_{t+1}\right) , \end{aligned}$$

10 $$\begin{aligned} \left[ {\mathop {{{\varvec{\mu }}}}\limits ^{{\tiny \rightarrow }}}_{\text {enc},t}^i, ({\mathop {{{\varvec{\sigma }}}}\limits ^{{\tiny \rightarrow }}}_{\text {enc},t}^{i})^2\right] _{i \in {\mathbb {I}}}&= \text {GN}_{\text {enc},{{\varvec{\phi }}}}\!\left( \left[ {{\varvec{x}}}_t, {\mathop {{{\varvec{h}}}}\limits ^{{\tiny \rightarrow }}}_{t-1} \right] \right)&\left[ {\mathop {{{\varvec{\mu }}}}\limits ^{{\tiny \leftarrow }}}_{\text {enc},t}^i, ({\mathop {{{\varvec{\sigma }}}}\limits ^{{\tiny \leftarrow }}}_{\text {enc},t}^{i})^2\right] _{i \in {\mathbb {I}}}&= \text {GN}_{\text {enc},{{\varvec{\phi }}}}\!\left( \left[ {{\varvec{x}}}_t, {\mathop {{{\varvec{h}}}}\limits ^{{\tiny \leftarrow }}}_{t+1} \right] \right) , \end{aligned}$$

11 $$\begin{aligned} \left[ {\mathop {{{\varvec{\mu }}}}\limits ^{{\tiny \rightarrow }}}_{\text {dec},t}^i, ({\mathop {{{\varvec{\sigma }}}}\limits ^{{\tiny \rightarrow }}}_{\text {dec},t}^{i})^2\right] _{i \in {\mathbb {I}}}&= \text {GN}_{\text {dec},{{\varvec{\theta }}}}\!\left( \left[ {\mathop {{{\varvec{z}}}}\limits ^{{\tiny \rightarrow }}}_t, {\mathop {{{\varvec{h}}}}\limits ^{{\tiny \rightarrow }}}_{t-1} \right] \right)&\left[ {\mathop {{{\varvec{\mu }}}}\limits ^{{\tiny \leftarrow }}}_{\text {dec},t}^i, ({\mathop {{{\varvec{\sigma }}}}\limits ^{{\tiny \leftarrow }}}_{\text {dec},t}^{i})^2\right] _{i \in {\mathbb {I}}}&= \text {GN}_{\text {dec},{{\varvec{\theta }}}}\!\left( \left[ {\mathop {{{\varvec{z}}}}\limits ^{{\tiny \leftarrow }}}_t, {\mathop {{{\varvec{h}}}}\limits ^{{\tiny \leftarrow }}}_{t+1} \right] \right) . \end{aligned}$$

Subsequent to their sampling in (8), the relative (delta) state updates, $\Delta {\hat{{{\varvec{x}}}}}_t$, are accumulated to produce predictions in absolute-space,

12 $$\begin{aligned} {\hat{{\mathop {{{\varvec{x}}}}\limits ^{{\tiny \rightarrow }}}}}_t&= {\mathop {{{\varvec{x}}}}\limits ^{{\tiny \rightarrow }}}_{t-1} + \Delta {\hat{{\mathop {{{\varvec{x}}}}\limits ^{{\tiny \rightarrow }}}}}_t&{\hat{{\mathop {{{\varvec{x}}}}\limits ^{{\tiny \leftarrow }}}}}_t&= {\mathop {{{\varvec{x}}}}\limits ^{{\tiny \leftarrow }}}_{t+1} + \Delta {\hat{{\mathop {{{\varvec{x}}}}\limits ^{{\tiny \leftarrow }}}}}_t. \end{aligned}$$

These predicted states ${\hat{{\mathop {{{\varvec{x}}}}\limits ^{{\tiny \rightarrow }}}}}_t$ and ${\hat{{\mathop {{{\varvec{x}}}}\limits ^{{\tiny \leftarrow }}}}}_t$ are then used to autoregressively update the next-timestep inputs using (1). The procedure then continues to autoregressively update the states for all timesteps *t* in each respective direction.

### Forward–backward fusion

The final directional outputs from the model are subsequently fused to produce the bidirectional estimates ${\hat{{{\varvec{x}}}}}^i_t$ for all agents. As in recent works on bidirectional LSTM-based imputation^42^, one method of fusion is to simply take the mean,

13 $$\begin{aligned} {\hat{{{\varvec{x}}}}}^i_t = 0.5\bigg ({\hat{{\mathop {{{\varvec{x}}}}\limits ^{{\tiny \rightarrow }}}}}^i_t + {\hat{{\mathop {{{\varvec{x}}}}\limits ^{{\tiny \leftarrow }}}}}^i_t\bigg ). \end{aligned}$$

Alternatively, at time *t*, let ${\mathop {\tau }\limits ^{{\tiny \rightarrow }}}^i_t$ and ${\mathop {\tau }\limits ^{{\tiny \leftarrow }}}^i_t$ denote the number of timesteps until the next ground truth observation in each direction, respectively. One can then weigh the contribution of each direction as,

14 $$\begin{aligned} {\hat{{{\varvec{x}}}}}^i_t = \bigg ({\mathop {\tau }\limits ^{{\tiny \rightarrow }}}^i_t {\hat{{\mathop {{{\varvec{x}}}}\limits ^{{\tiny \rightarrow }}}}}^i_t + {\mathop {\tau }\limits ^{{\tiny \leftarrow }}}^i_t {\hat{{\mathop {{{\varvec{x}}}}\limits ^{{\tiny \leftarrow }}}}}^i_t\bigg )\,\bigg ({\mathop {\tau }\limits ^{{\tiny \rightarrow }}}^i_t + {\mathop {\tau }\limits ^{{\tiny \leftarrow }}}^i_t\bigg )^{-1}. \end{aligned}$$

This ensures predictions corresponding to the direction with the most recent observation are weighted higher. For example, if at prediction timestep *t*, the nearest ground truth observations in the future and past for agent *i* occur at $t+8$ and $t-2$, then ${\mathop {\tau }\limits ^{{\tiny \rightarrow }}}^i_t = 8$ and ${\mathop {\tau }\limits ^{{\tiny \leftarrow }}}^i_t = 2$, such that ${\hat{{{\varvec{x}}}}}^i_t = 0.8{\hat{{\mathop {{{\varvec{x}}}}\limits ^{{\tiny \rightarrow }}}}}^i_t + 0.2{\hat{{\mathop {{{\varvec{x}}}}\limits ^{{\tiny \leftarrow }}}}}^i_t$.

### Training

As in prototypical VAE pipelines, we update model parameters in each iteration of the algorithm by maximizing the evidence lower bound (ELBO) over all the agents in each trajectory,

15 $$\begin{aligned}&\sum _{t\in {\mathbb {T}}} \bigg [ \mathbb {E}_{ q_{{{\varvec{\phi }}}} ({\mathop {{{\varvec{z}}}}\limits ^{{\tiny \rightarrow }}}_t | {{\varvec{x}}}_t, {\mathop {{{\varvec{x}}}}\limits ^{{\tiny \rightarrow }}}_{< t}, {\mathop {{{\varvec{z}}}}\limits ^{{\tiny \rightarrow }}}_{<t} ) }\left[ \log p_{{{\varvec{\theta }}}}\bigg (\Delta {\hat{{\mathop {{{\varvec{x}}}}\limits ^{{\tiny \rightarrow }}}}}_t | {\mathop {{{\varvec{x}}}}\limits ^{{\tiny \rightarrow }}}_{<t}, {\mathop {{{\varvec{z}}}}\limits ^{{\tiny \rightarrow }}}_{\le t}\bigg )\right] - \beta D_{KL}\bigg ( q_{{{\varvec{\phi }}}}\bigg ({\mathop {{{\varvec{z}}}}\limits ^{{\tiny \rightarrow }}}_t | {{\varvec{x}}}_t, {\mathop {{{\varvec{x}}}}\limits ^{{\tiny \rightarrow }}}_{< t}, {\mathop {{{\varvec{z}}}}\limits ^{{\tiny \rightarrow }}}_{<t}\bigg ) || p_{{{\varvec{\theta }}}}\bigg ({\mathop {{{\varvec{z}}}}\limits ^{{\tiny \rightarrow }}}_t | {\mathop {{{\varvec{x}}}}\limits ^{{\tiny \rightarrow }}}_{<t}, {\mathop {{{\varvec{z}}}}\limits ^{{\tiny \rightarrow }}}_{<t}\bigg )\bigg ) \nonumber \\&\quad + \mathbb {E}_{ q_{{{\varvec{\phi }}}} ({\mathop {{{\varvec{z}}}}\limits ^{{\tiny \leftarrow }}}_t | {{\varvec{x}}}_t, {\mathop {{{\varvec{x}}}}\limits ^{{\tiny \leftarrow }}}_{> t}, {\mathop {{{\varvec{z}}}}\limits ^{{\tiny \leftarrow }}}_{>t} ) }\left[ \log p_{{{\varvec{\theta }}}}\bigg (\Delta {\hat{{\mathop {{{\varvec{x}}}}\limits ^{{\tiny \leftarrow }}}}}_t | {\mathop {{{\varvec{x}}}}\limits ^{{\tiny \leftarrow }}}_{>t}, {\mathop {{{\varvec{z}}}}\limits ^{{\tiny \leftarrow }}}_{\ge t}\bigg ) \right] - \beta D_{KL}\bigg ( q_{{{\varvec{\phi }}}}\bigg ({\mathop {{{\varvec{z}}}}\limits ^{{\tiny \leftarrow }}}_t | {{\varvec{x}}}_t, {\mathop {{{\varvec{x}}}}\limits ^{{\tiny \leftarrow }}}_{> t}, {\mathop {{{\varvec{z}}}}\limits ^{{\tiny \leftarrow }}}_{>t}\bigg ) || p_{{{\varvec{\theta }}}}\bigg ({\mathop {{{\varvec{z}}}}\limits ^{{\tiny \leftarrow }}}_t | {\mathop {{{\varvec{x}}}}\limits ^{{\tiny \leftarrow }}}_{>t}, {\mathop {{{\varvec{z}}}}\limits ^{{\tiny \leftarrow }}}_{>t}\bigg )\bigg ) \bigg ], \end{aligned}$$

where $\beta$ is a weighing term on the VAE KL-regularizer^44^. For training, we maximize (15) over mini-batches of trajectories sampled from our dataset.

In our experiments, we also consider several ablations of the models, including: decoders that take as input only the latent states ${\mathop {{{\varvec{z}}}}\limits ^{{\tiny \rightarrow }}}_t$ and ${\mathop {{{\varvec{z}}}}\limits ^{{\tiny \leftarrow }}}_t$ (i.e., disabling the *skip-connection* from the LSTM hidden states ${\mathop {{{\varvec{h}}}}\limits ^{{\tiny \rightarrow }}}_{t-1}$ and ${\mathop {{{\varvec{h}}}}\limits ^{{\tiny \leftarrow }}}_{t+1}$ to the decoder in (11)); and *next-step conditioned* graph-decoders that include nodes with features ${{\varvec{v}}}^{i}$ locked to agent observations available for the timestep being predicted (i.e., observed decoder nodes with features ${{\varvec{x}}}^i_t \odot {{\varvec{m}}}^i_t$, which only send messages during message-passing, and thus do not update their states at prediction timestep *t* as they are observable).

### Sweeps and hyperparameters

We conduct a wide hyperparameter sweep and report the results corresponding to the best hyperparameters for each model. We train for $10^5$ iterations, with a batch size of 64 trajectories, using the Adam optimizer^45^ with a learning rate of 0.001 (and default exponential decay parameters, $b_1=0.9$, and $b_2=0.999$). For LSTM-based models (including the ones used in the Graph Imputer), we use 2-layer LSTMs with 64 hidden units each. For the graph edge and node update networks, $f_{{{\varvec{\theta }}}}^e$ and $f_{{{\varvec{\theta }}}}^v$, we use 2-layer MLPs with 64 hidden units each, with internal ReLU activations^46^. In the ELBO (15), we anneal $\beta$ from an initial value of 0.1 to final values of 0.01 and 1 in our sweeps. All variational models use 16-dimensional latent variables ${{\varvec{z}}}$. For all bidirectional models, we sweep over the two fusion modes specified in (13) and (14). For each model, training for each hyperparameter set is conducted and reported over a sweep of 5 random seeds. Additional hyperparameters and computational resources used are detailed in the Additional Experiment Details section of the Supplementary Information.

### Camera model details

Given the camera model described in the main text, on average, $12.76 \pm 3.70$ players (out of 22) are in-frame in each sequence, with a consecutive in-frame duration of $4.94s \pm 3.49$ s. Under this camera model, certain players are at times completely out of view for the entire trajectory duration. To provide some warm-up context to the models during training, we include an additional 5 frames of observations at the beginning and end of all trajectories for all players. In practical evaluation settings involving longer trajectory sequences, the camera pans around such that all players are effectively observed at some stage, thus not requiring this.

### Pitch control model

Pitch control^14^ is a technique for quantifying a football team’s level of ball control, throughout the pitch. At a high level, the model takes as input the state of play (i.e., the positions and velocities of the players and the ball), and computes the probability of each player being able to gain control of the ball were it to be passed to every location on pitch; in practice, the pitch is discretized such that these probabilities are computed over a finite number of locations (we use a 60 $\times$ 40 discretization of the pitch in our experiments, as this provides a fairly smooth view of the pitch control field without incurring significant computational expense).

In more detail, the pitch control model relies on a physics-based motion model, incorporating factors such as ball time-of-flight and player time-to-intercept to compute control probabilities. In our experiments, we use a slightly simplified variant, wherein at any given timestep in a trajectory we consider the ball being passed to each discrete pitch location under a constant reference speed (as detailed later). Subsequently, each player on the pitch is assumed to travel in a straight line to the ball’s target destination at a reference top speed. Following these simplifications, we use the model detailed in Spearman et al.^14^ to compute the player-wise pitch control probabilities. Next, to compute team-wise pitch control field, we simply sum up player-wise pitch control per team and compute the difference between the resulting scalar fields for home and away team. Both the ball and player reference speeds are fit to our data, by first computing all player and ball velocities throughout the dataset, and subsequently choosing the 75th percentile speed for each (in practice, we found the Pitch Control model to be fairly insensitive to the specific percentile chosen, as long as the ratio between ball and player speeds remained similar).

## Supplementary Information

Supplementary Information.

## Data Availability

The datasets generated and/or analyzed during the current study are not publicly available due to licensing restrictions. However, contact details of the data providers are available from the corresponding authors on reasonable request.
