# Supplementary material for: Multiagent off-screen behavior prediction in football
Source: Sci Rep. 2022 May 23;12:8638. doi: 10.1038/s41598-022-12547-0 (PMC9126960; doi:10.1038/s41598-022-12547-0)
Supplement: Supplementary file 1 — Supplementary Information. [file 41598_2022_12547_MOESM1_ESM.pdf]

Supplementary Information: Multiagent Off-screen Behavior Prediction in Football

We provide here supplementary information that may be of interest to the reader. Note that sections and figures in the main text that are referenced here are clearly indicated via numerical counters (e.g., Fig. 1), whereas those in the appendix itself are indicated by alphabetical counters (e.g., Fig. S1).

Additional Experiment Details

Baseline Model Details

This section details the Role-invariant VRNN baselines presented in Table 1 of the main paper. As mentioned earlier, this model is designed specifically for the case of football, assuming that each trajectory stream consists of two teams (where permutation-invariance to player ordering is desired within each) and the ball.

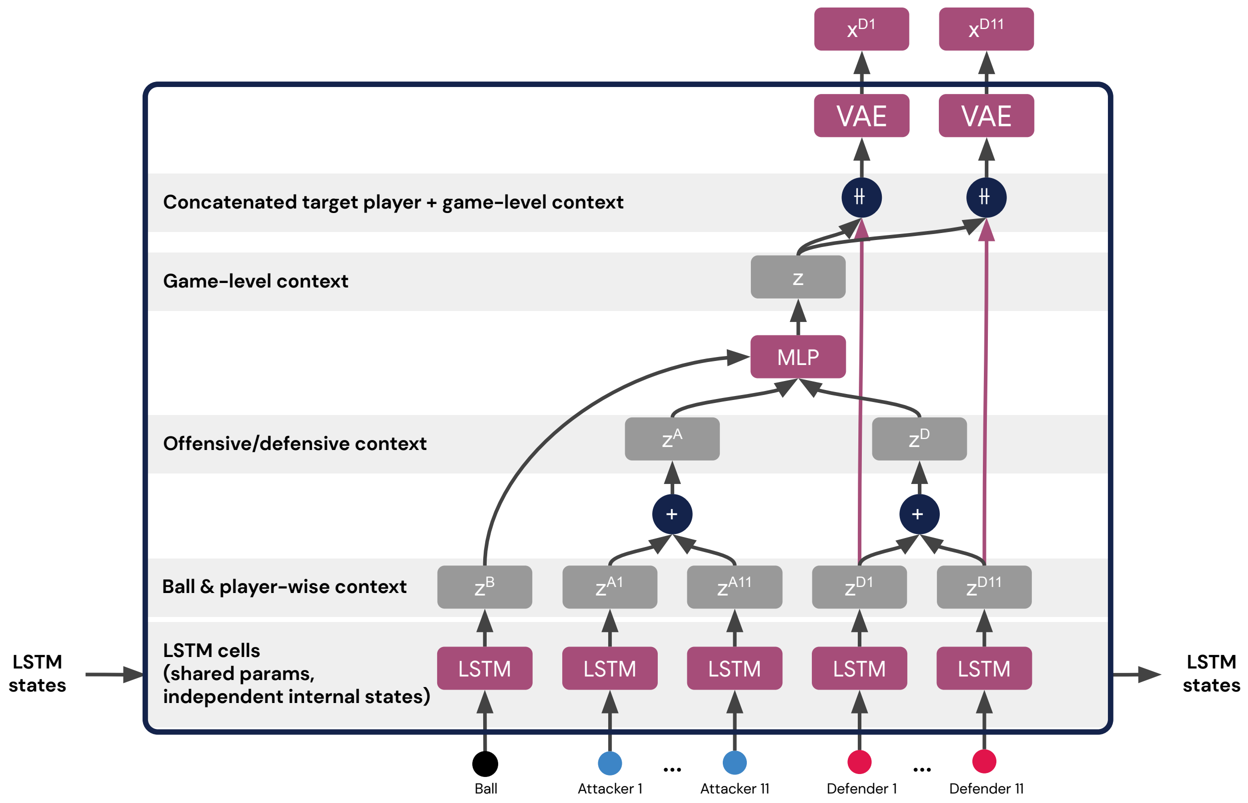

Figure S1: Football-specific role-invariant VRNN baseline architecture. The input manipulations conducted in this model, which are detailed in the Baseline Model Details section of the Supplementary Information, ensure invariance of outputs to permutations of player orders within each time. However, this also implies the strongest assumption on the domain at hand, which is interaction of two teams of players, along with a singleton entity (the ball), in a shared environment.

We provide an overview of this model in Fig. S1. At each timestep, this model works by passing the player and ball observations through LSTMs with shared parameters. The hidden states from the LSTMs are subsequently summed up within each team, thus producing team-level contextual vectors; note that this operation ensures permutation-invariance within each team. Subsequently, the ball and each team's hidden state are passed through an MLP, thus producing a vector representing the overall game-level context. Finally, to produce predictions for individual players, this game-level context is concatenated to their individual

LSTM states to produce a player-specific context, which is then passed through an MLP-based VAE, enabling sampling of players' next-states.

The process iterates autoregressively as in the Graph Imputer, and likewise can be repeated in reverse temporal fashion to produce and fuse bidirectional player state estimates. Training of this model is conducted in the same manner as the Graph Imputer, using the ELBO (15).

In addition to this model, we also include in Table S2 a variant called 'Role-invariant RNN', which simply replaces the VAE head in Fig. S1 with an MLP.

Additional Hyperparameter Details & Computational Resources

In addition to the key hyperparameters detailed in the main paper, we also ran sweeps for VAE-based models wherein a standard normal prior distribution was used (in lieu of a learned prior), as typically also considered in VAE approaches. For the Social LSTM model, we also ran sweeps over grid widths 8, 24, and 64 capturing the size of neighbor grids for each player (in meters); larger grid sizes correspond to increasing amounts of neighbor context on the football pitch.

For training, we use a cluster of Tesla V100 and P100 GPUs for training and evaluation, respectively. Overall, our sweeps were conducted over a set of 435 independent training runs (i.e., each with a unique hyperparameter set and random seed). Depending on the simplicity of the underlying model (simplest being the autoregressive LSTM, and most complex being the Graph Imputer), each training run took approximately 3 to 15 hours of wallclock time to train.

Additional Experiment Results

Additional Sweeps and Baselines

Table S2 presents additional comparative sweeps for the football off-screen player state estimation scenario. In addition to the results in the main paper, this table includes the Role-invariant RNN baseline detailed in the Baseline Model Details section. The Role-invariant RNN model achieves quite similar performance as the Role-invariant VRNN counterpart, with the main distinction being that the former model is deterministic, in contrast to the latter; in certain applications, the ability to resample the model (or, e.g., fine-tune the KL-regularization β in (15) to increase or decrease the level of stochasticity in the model) can be quite useful from a practical perspective.

Additionally, Table S2 includes sweeps over the bidirectional fusion modes ((13) and (14) in the Methods section of the main text). For all bidirectional models, we observe that the nearest-observation weighted fusion mode (14) yields the lowest evaluation loss, primarily as it modulates the weighting of the directional updates (which deviate from the ground truth the longer they have not made an observation).

Sensitivity to Observability Model

Table S1: Comparison of model performance under increased partial observability. Here we decrease the simulated camera's (horizontal, vertical) field-of-view from (45°, 30°) to (30°, 20°), effectively decreasing the average number of visible players in each frame by 30%.

Model	$\mathcal{L}_2$(Mean)
Spline (Linear)	1.30 ± 0.117
Spline (Quadratic)	0.384 ± 0.036
Spline (Cubic)	0.376 ± 0.036
Social LSTM [7]	1.607 ± 0.039
Bidir. Social LSTM	0.342 ± 0.013
GVRNN [12]	1.487 ± 0.023
Graph Imputer (Ours)	**0.302 ± 0.005**

Table S2: Football off-screen player state estimation results. We separate models into two categories: restricted models (those that apply only to the football setting, as they process data in a manner explicitly assuming two teams of players, along with a ball), and general models (models that apply to general multiagent prediction settings). The columns refer to the following: **Skip connection**: whether a skip-connection from the input to the decoder is enabled for autoencoder based models. **Next-step conditional decoder**: whether decoders in graph network-based models condition on available next-timestep observations, as additional context. **Bidir. fusion mode**: the fusion mode used for bidirectional models, where 'mean' corresponds to (13) in the main text, and 'nearest' to (14). For each baseline model, we compute the mean evaluation loss, $\mathcal{L}_2$(Mean), compared to the ground truth trajectories (over all seeds). For stochastic models, for each evaluation sequence we also take 6 samples of imputed trajectories, and also report the minimum evaluation loss, $\mathcal{L}_2$(Min.), over all samples, averaged over all seeds.

	Model	Skip connection	Next-step conditional decoder	Bidirectional fusion mode	$\mathcal{L}_2$(Mean)	$\mathcal{L}_2$(Min.)
Restricted	Role-invariant RNN	–	–	–	0.940 ± 0.01	–
	Bidir. Role-invariant RNN	–	–	Mean	0.442 ± 0.004	–
	Bidir. Role-invariant RNN	–	–	Nearest	0.164 ± 0.004	–
	Role-invariant VRNN	✗	–	–	2.020 ± 2.03	1.960 ± 2.063
	Role-invariant VRNN	✓	–	–	0.958 ± 0.009	0.953 ± 0.009
	Bidir. Role-invariant VRNN	✗	–	Mean	0.510 ± 0.02	0.486 ± 0.019
	Bidir. Role-invariant VRNN	✗	–	Nearest	0.174 ± 0.002	0.160 ± 0.002
	Bidir. Role-invariant VRNN	✓	–	Mean	0.456 ± 0.009	0.455 ± 0.008
	Bidir. Role-invariant VRNN	✓	–	Nearest	0.167 ± 0.002	0.166 ± 0.002
General	Spline (Linear)	–	–	–	0.658 ± 0.081	–
	Spline (Quadratic)	–	–	–	0.197 ± 0.023	–
	Spline (Cubic)	–	–	–	0.193 ± 0.023	–
	LSTM	–	–	–	1.579 ± 0.019	–
	Bidir. LSTM	–	–	Mean	0.751 ± 0.009	–
	Bidir. LSTM	–	–	Nearest	0.350 ± 0.006	–
	Social LSTM[7]	–	–	–	1.049 ± 0.274	–
	Bidir. Social LSTM	–	–	Mean	0.457 ± 0.011	–
	Bidir. Social LSTM	–	–	Nearest	0.198 ± 0.052	–
	GVRNN[12]	✗	✗	–	2.243 ± 0.136	1.453 ± 0.073
	GVRNN[12]	✗	✓	–	2.447 ± 1.197	2.400 ± 1.231
	GVRNN[12]	✓	✗	–	0.882 ± 0.009	0.874 ± 0.009
	GVRNN[12]	✓	✓	–	0.865 ± 0.018	0.852 ± 0.017
	Graph Imputer (Ours)	✗	✗	Mean	0.666 ± 0.087	0.638 ± 0.09
	Graph Imputer (Ours)	✗	✗	Nearest	0.241 ± 0.05	0.224 ± 0.051
	Graph Imputer (Ours)	✗	✓	Mean	1.106 ± 0.368	1.094 ± 0.381
	Graph Imputer (Ours)	✗	✓	Nearest	0.404 ± 0.102	0.397 ± 0.11
	Graph Imputer (Ours)	✓	✗	Mean	0.452 ± 0.041	0.449 ± 0.04
	Graph Imputer (Ours)	✓	✗	Nearest	0.165 ± 0.005	0.163 ± 0.005
	Graph Imputer (Ours)	✓	✓	Mean	0.418 ± 0.005	0.414 ± 0.005
	Graph Imputer (Ours)	✓	✓	Nearest	**0.153 ± 0.003**	**0.151 ± 0.003**

As mentioned earlier, we anticipate that situations with increased partial observability will further compound errors associated with with standard interpolation techniques. To further investigate this, we generated a new dataset, reducing the camera's (horizontal, vertical) field-of-view from (45°, 30°) to (30°, 20°). Under this new camera model, on average, 8.51 ± 3.36 players (out of 22) are in-frame in each sequence, with a consecutive in-frame duration of $3.76s \pm 3.09s$; for comparison, these quantities were respectively 12.76 ± 3.70 players in-frame and $4.94s \pm 3.49s$ in the original dataset reported in the main paper, thus illustrating a notable decrease in observability. Retraining the models with this new dataset results in the performance metrics reported in Table S1 (which are shown for the best hyperparameters for each model). In this new setting involving players that are out-of-view for longer periods of time, we see that the bidirectional social LSTM now outperforms the spline-based baselines. However, our Graph Imputer model continues to also substantially outperform all models, which provides further evidence of the robustness of our approach.

Additional Trajectory Visualizations

We provide a number of additional visualizations of trajectory predictions for the Graph Imputer and additional baselines in Figs. S2 to S5. Here we also include a variant of the Graph Imputer which attains high trajectory sequence variance, which can be useful from a downstream analytical perspective when higher sample stochasticity is desired.

Additional Details on Related Works

Table S3 provides an additional cross-section overview of the most closely related works to ours. In this table, we summarize models that consider prediction of trajectories, detailing whether or not they are stochastic, consider the interactions of multiple agents in the system, target the imputation problem (as opposed to the typical forward-prediction setting), and use both forward- and backward-information. Some of the models in this table are related to ours, although target slightly different problem regimes. For example, in Naomi [39], the considered dataset regime is distinct from ours in that they consider scenarios wherein at each timestep either all players are simultaneously observed, or all are unobserved. By contrast, we consider situations where *a subset* of players is observed (while others are unobserved) at any given timestep; this is the scenario encountered in the off-screen player tracking problem targeted herein, where some players may be visible on-screen, whereas others may be off-screen. This distinction enables the approach of Naomi to essentially treat the multiagent observation $\boldsymbol{x}_t$ at each time t as a single, high-dimensional input. Indeed, that is the primary distinction from our graph network-based approach, where the decompositionality afforded by the graph structure enables our model to treat mixed-observability settings. Similarly, in Baller2vec++ [30], the introduced model uses multiagent information, though does not target the imputation setting considered here (where we consider distant future observations that are available for a subset of agents, with heterogeneous temporal gaps in observed data). The key contribution of their work, rather, is to use the probability chain rule to condition the generated trajectories of one agent on the generated trajectories of other agents, to induce better-correlated predictions, which is indeed an important feature to capture in sports-based models.

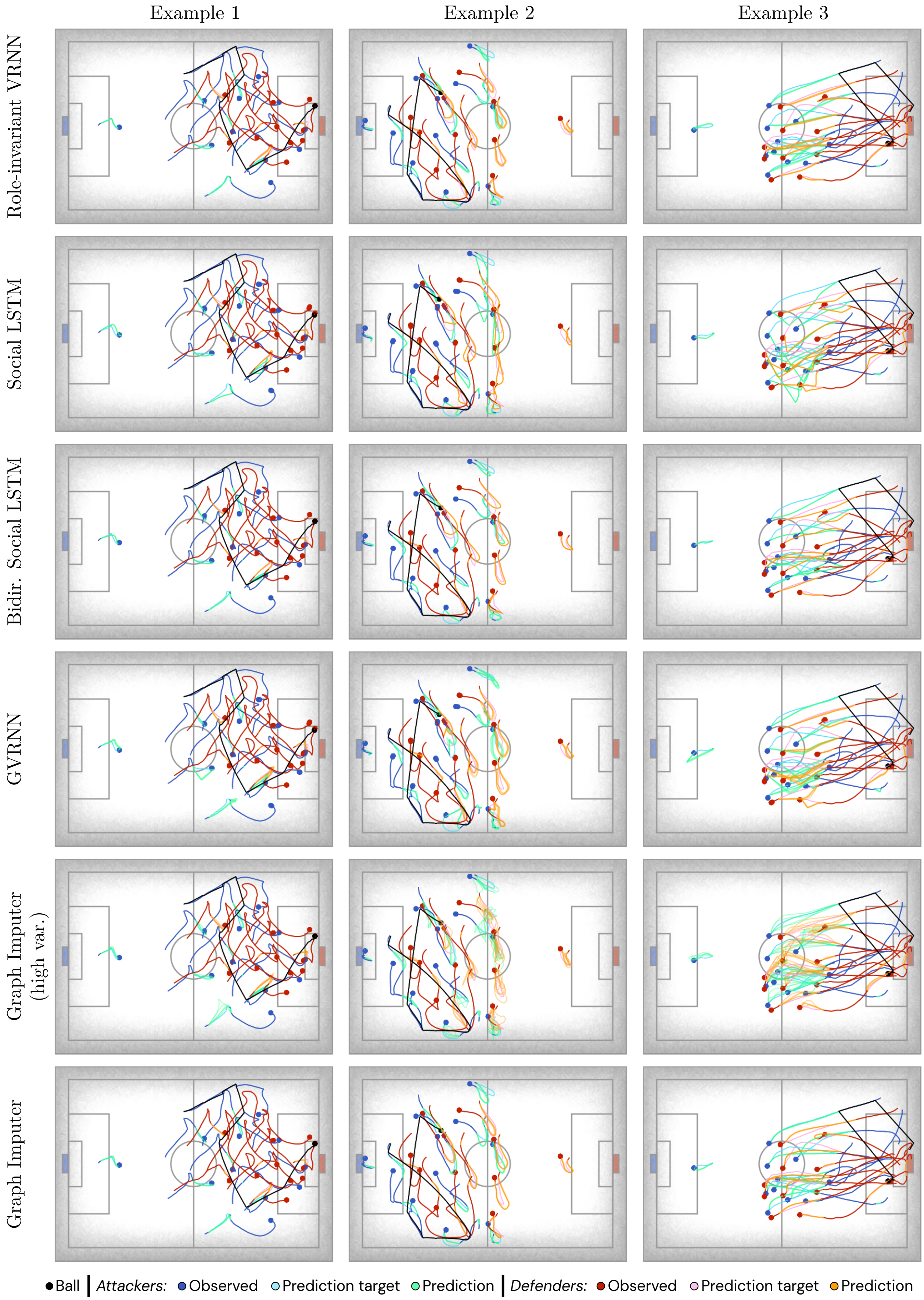

Figure S2: Trajectory visualizations (best viewed when zoomed in). Each column provides an example trajectory sequence, with rows showing results from various models, including the Graph Imputer (ours).

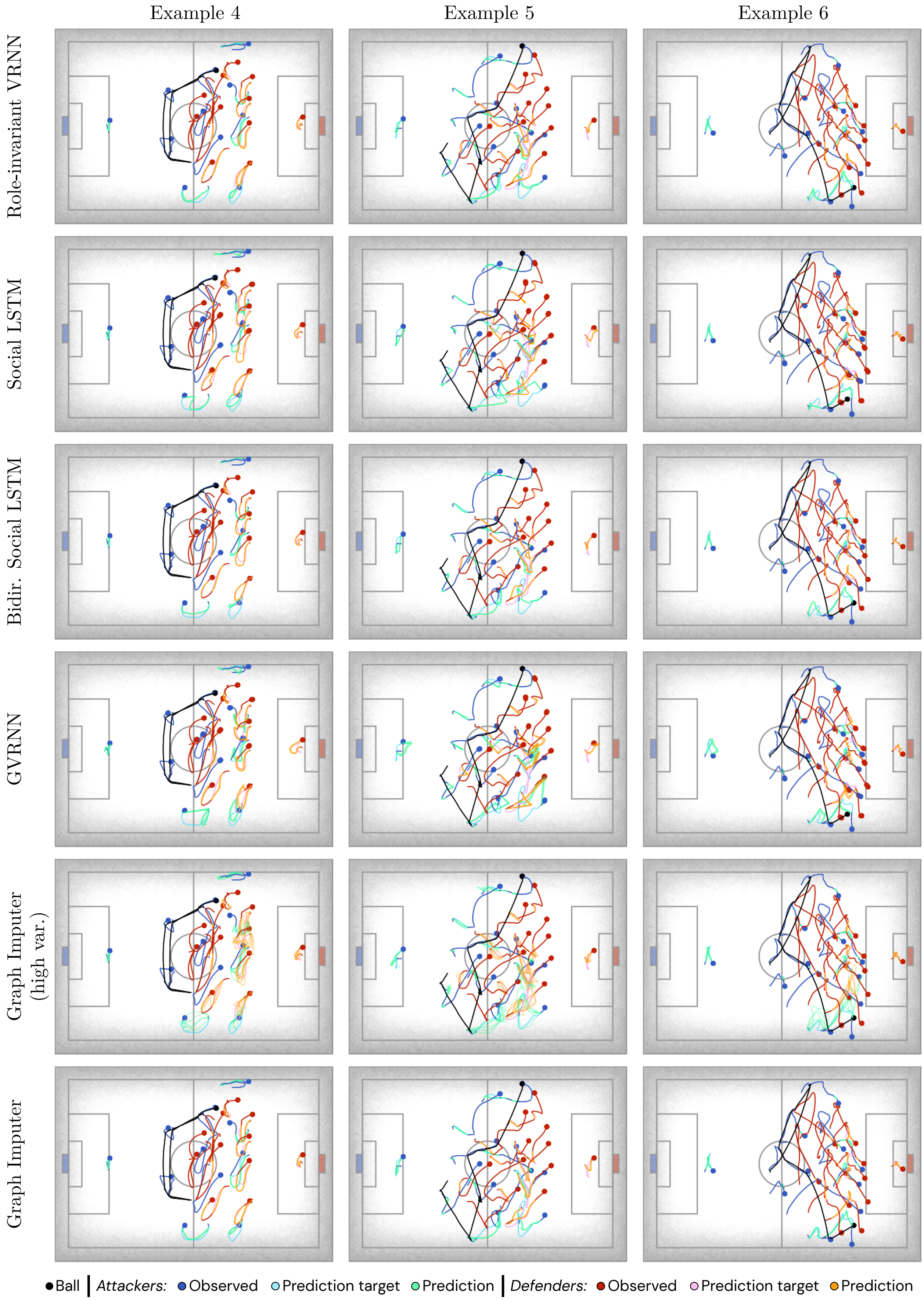

Figure S3: Trajectory visualizations (best viewed when zoomed in). Each column provides an example trajectory sequence, with rows showing results from various models, including the Graph Imputer (ours).

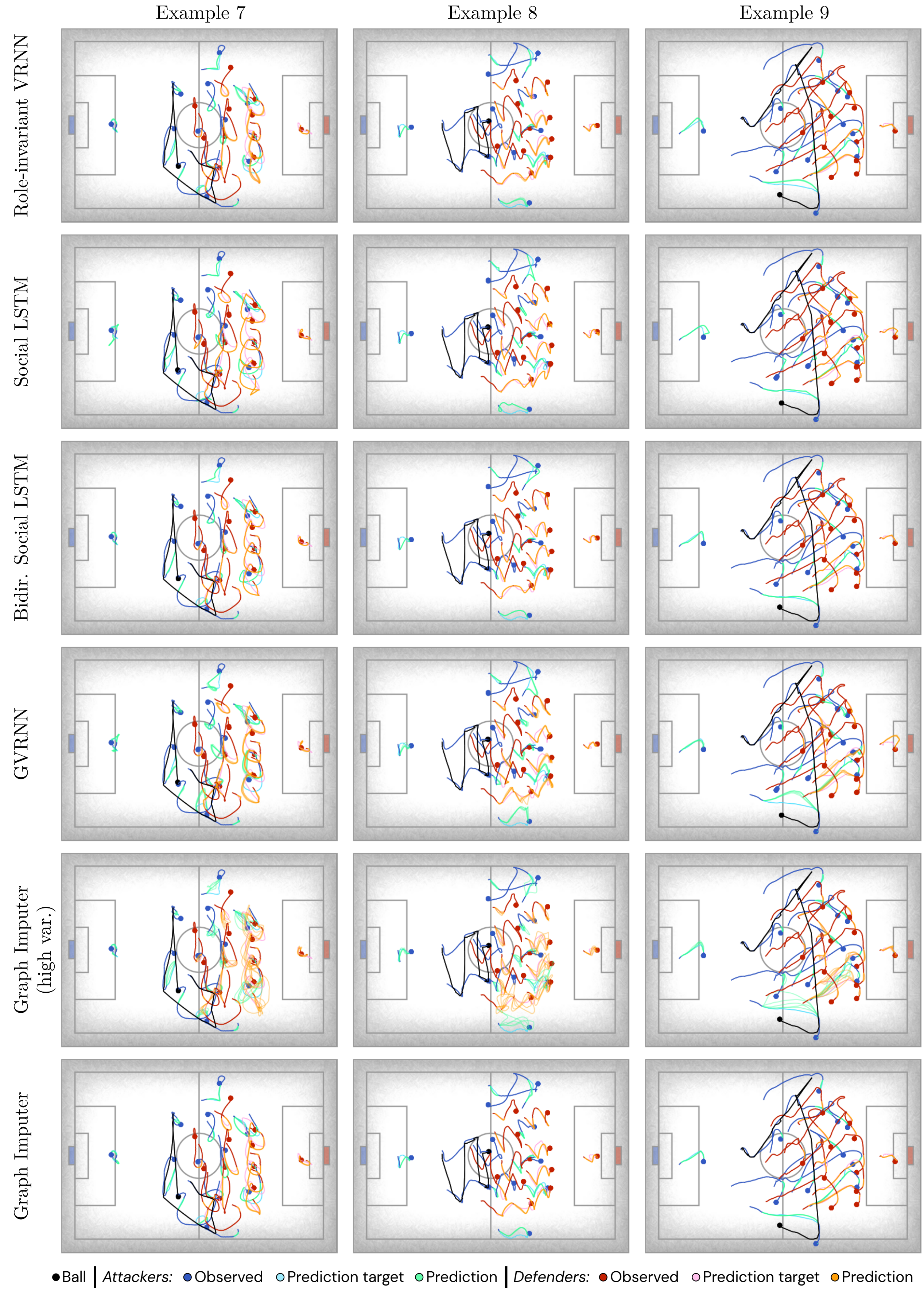

Figure S4: Trajectory visualizations (best viewed when zoomed in). Each column provides an example trajectory sequence, with rows showing results from various models, including the Graph Imputer (ours).

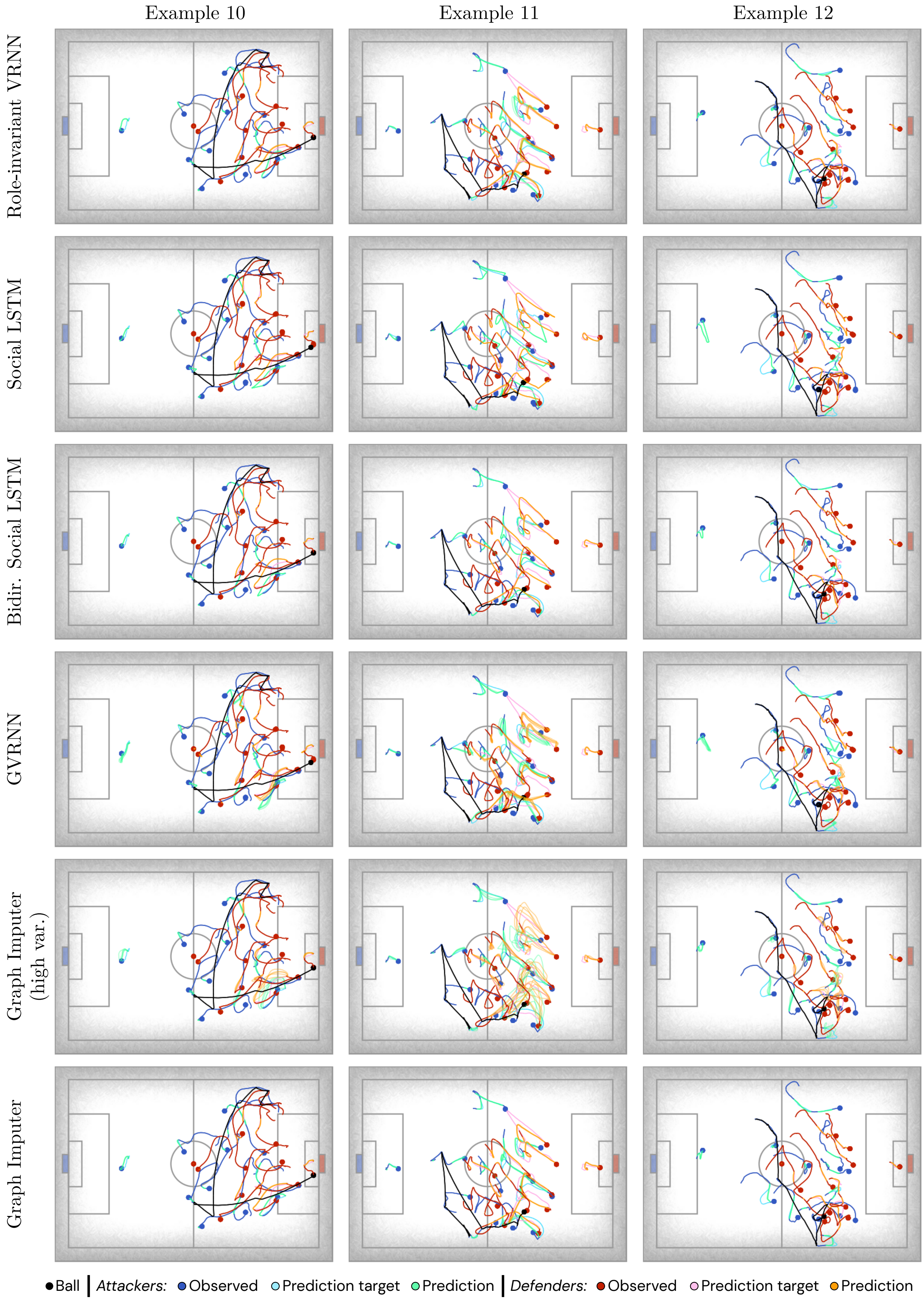

Figure S5: Trajectory visualizations (best viewed when zoomed in). Each column provides an example trajectory sequence, with rows showing results from various models, including the Graph Imputer (ours).

Table S3: Overview of the attributes of the different models covered in our related work section, in comparison to our Graph Imputer model. In this table, we summarize models that consider prediction of trajectories, detailing whether or not they are stochastic, consider the interactions of multiple agents in the system, target the imputation problem (as opposed to the typical forward-prediction setting), and use both forward- and backward-information.

	Human trajectories	Stochastic	Considers interactions between agents	Imputation	Forward & backward information
Time series classification [38]	✗	✗	✗	✓	✗
BRITS [42]	✗	✗	✓ Weighted conditioning	✓	✓
M-RNN [43]	✗	✗	✗	✓	✓
VAIN [15]	✗ No seq. pred.	✗	✓Interaction nets	✗	✗
Volleyball trajectory prediction [18]	✗ Just the ball	✗	✗	✗	✗
GAN models [40,41]	✗	✓	✓ Conditioning	✓	✗
Social LSTM [7]	✓ Pedestrians	✗	✓ Social pooling layer	✗	✗
MBT [13]	✓ Basketball	✓	✗	✗	✗
Imitation Learning [10,11]	✓ Football	✓	✓ Conditioning	✗	✗
Reciprocal learning nets [29]	✓ Pedestrians	✓	✓ Social pooling layer	✗	✓(As regularization)
EvolveGraph [19]	✓ Basketball	✓	✓	✗	✗
GraphVRNN [12]	✓ Basketball & Football	✓	✓	✗	✗
Naomi [39]	✓ Basketball	✓	✗	✓	✓
NRI [36]	✓ Basketball	✓	✓	✗	✗
Baller2vec [16]	✓ Basketball	✓	✓	✗	✗
Baller2vec++ [30]	✓ Basketball	✓	✓	✗	✓
Graph Imputer (Ours)	✓ Football	✓	✓	✓	✓
